# Vitamin D deficiency in non-autoimmune hypothyroidism: a case-control study

**DOI:** 10.1186/s12902-020-0522-9

**Published:** 2020-03-20

**Authors:** Salma Ahi, Mohammad Reza Dehdar, Naser Hatami

**Affiliations:** 1 Research Center for Noncommunicable Diseases, Jahrom University of Medical Sciences, Jahrom, Iran; 2 Student Research Committee, Jahrom University of Medical Sciences, Jahrom, Iran

## Abstract

**Background:**

Although in many studies, the relationship between autoimmune hypothyroidism (Hashimoto) and Vitamin D deficiency was shown, no research has been performed on the role of vitamin D in non-autoimmune hypothyroidism.

**Methods:**

This was a case-control study in Endocrinology clinic of Jahrom (south of Iran). The patients with Hashimoto (*n* = 633) and non-Hashimoto hypothyroidism (*n* = 305), along with a control group (*n* = 200) were evaluated. 25(OH) D level, T3 and T4 levels were studied and Anti TPO and Anti TG tests were performed. The results of vitamin D level were analyzed and interpreted using SPSS in terms of the cause of hypothyroidism (immune and non-immune).

**Results:**

The results of the study showed a significantly lower level of vitamin D in both immune and non-immune Hashimoto’s thyroiditis (HT) in comparison to healthy controls (*P* < 0.05). We observed a significant inverse correlation between the vitamin D and TGAb level (*p* = 0.001, r = − 0.261) and a direct correlation of vitamin D with TSH level (*p* = 0.008, r = 0.108) in Hashimoto thyroiditis patients.

**Conclusion:**

Finally, the results indicated that non-autoimmune hypothyroidism, as well as HT, is associated with vitamin D deficiency. The role of vitamin D deficiency in Hashimoto thyroiditis was thought to be in the association of higher autoantibody (TGAb) level; while, there should be further studies determining vitamin D deficiency’s role in non-immune hypothyroidism.

## Background

Vitamin D receptors exist in many body organs. Through these receptors, vitamin D has various functions, including the regulation of ion homeostasis, cell growth, cell differentiation, and cellular immunity [1]. Vitamin D plays an important role in preventing the occurrence of many inflammatory diseases, infections, and autoimmune diseases [2]. In numerous studies, the relationship between vitamin D deficiency and a variety of diseases, including musculoskeletal [3], cardiovascular [4], kidney disease [5], diabetes [6] and infections [7] had been shown. The thyroid gland is also one of the organs that have a receptor for vitamin D. The vitamin D receptor in the thyroid is a member of a large group of receptors called nuclear receptors, which also belong to the thyroid hormones receptor [8]. Some studies indicated that vitamin D deficiency is associated with various autoimmune diseases [9]. Today, Hashimoto is one of the most common acquired hypothyroidism and autoimmune disease in children and adults [10]. The onset of autoimmune-thyroid disease with vitamin D deficiency is very common [11]. Plenty of evidence has shown the role of vitamin D in the regulation of pro-inflammatory cytokines, regulatory T cell, and immune response [12]. It seems that vitamin D deficiency leads to an increase in the risk of autoimmune diseases. Vitamin D also can reduce the pathogenesis of DCs cells, macrophage, CD4 + T, CD8 + T, and B cells [9]. Besides, it has been shown as a selective immune inhibitor that plays an important role in suppressing and preventing the development of autoimmune diseases such as encephalopathy, rheumatoid arthritis, systemic lupus erythematosus, diabetes type 1, and intestinal inflammatory diseases [13–15]. Recent studies have shown the role of vitamin D deficiency in autoimmune thyroids, such as Hashimoto thyroiditis [16, 17]. To the best of our knowledge, there is contradictory research about the relationship between thyroid diseases, especially hypothyroidism and vitamin D deficiency; therefore, in the present study, we aimed to evaluate the vitamin D level in hypothyroidism patients. Besides, there was no study comparing the vitamin D level in immune and non-immune hypothyroidism, and the relationship between the anti-TPO level and vitamin D, as well as the disease treatment status and vitamin D; hence, in the present study we evaluated the mentioned issues.

## Method

### Study size and participants

**Fig. 1:**
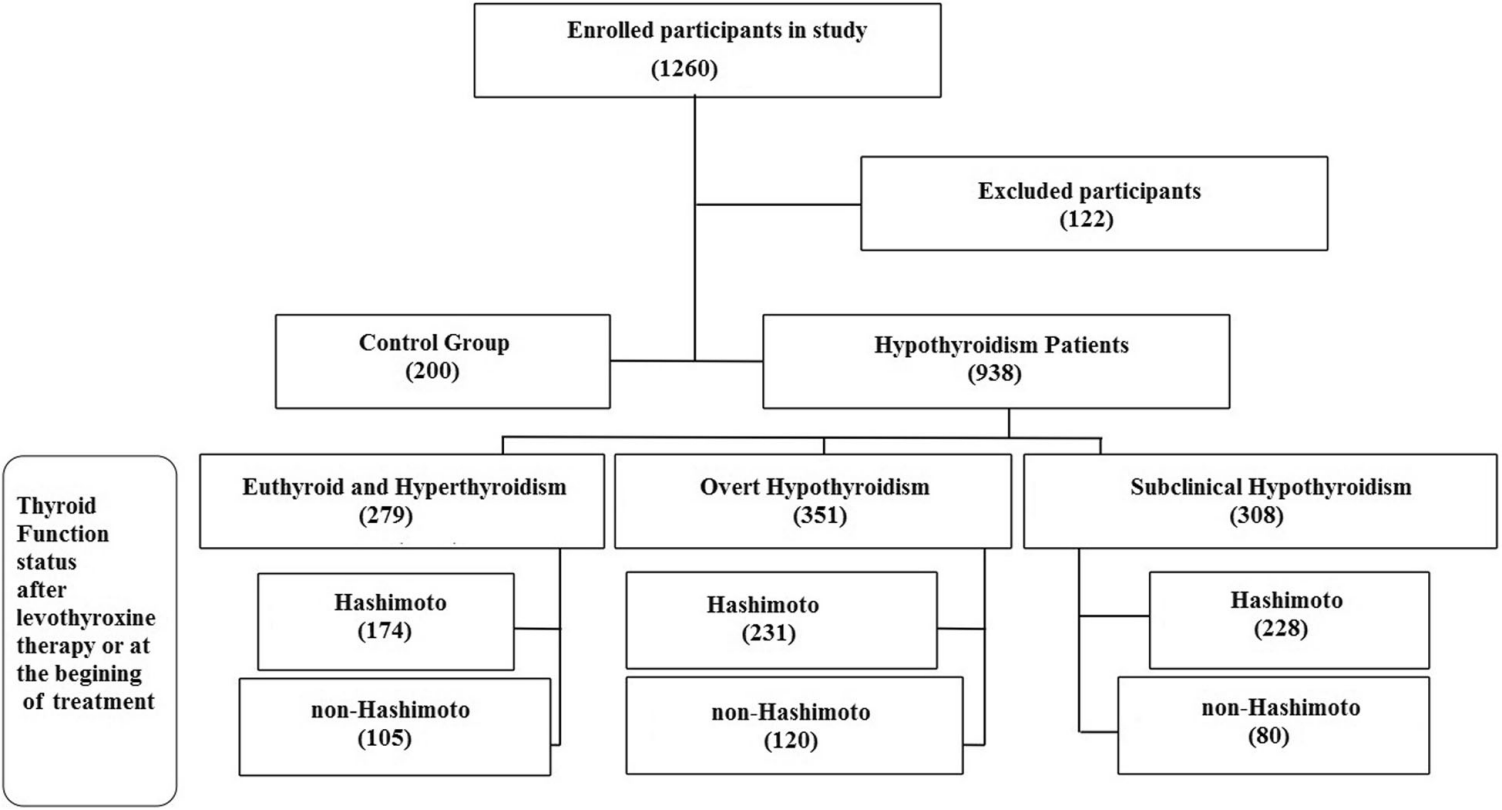
Study participants. Included and excluded Study participants with thyroid function status of enrolled hypothyroidism patients as Case group

In the present cross-sectional case control study, all hypothyroidism patients were selected among referents to the endocrinology clinic of Jahrom city in 2018. In the study, the treatment group included patients with pre-diagnosed hypothyroidism under Levothyroxine therapy or newly diagnosed patients. Written informed consent was acquired from all study subjects. Control group consisted of healthy people, who were similar to other groups in terms of confounding variables. Exclusion criteria were to have collagen vascular and Celiac disease and type-1 diabetes mellitus. Finally, 633 Immune Hypothyroid and 305 non-Immune Hypothyroid and 200 healthy subjects were enrolled (Fig. 1). The exclusion criteria for this study were type 1 diabetes, lupus, collagen vascular disease, rheumatoid arthritis, celiac disease, and also patients who underwent vitamin D supplementation and vitamin D interacting medications (such as antacids, corticosteroids, orlistat, diabetes medications, antihypertensive drugs, cholestyramine, antiepileptics, calcium supplements). While the participants enrolment were done based on their previous thyroid function workups during referral to thyroid clinic, to investigate the disease and vitamin D interactions, new blood samples were taken to evaluate both vitamin D levels and thyroid function tests in same time at last month of summer with adequate sun exposure. Because, the circulating vitamin D levels ranges from season to season, in our study all blood samples were taken at the same period of august 2018; so, the confounding effect of seasonal variations of vitamin D was eliminated.

### Outcome definition

Normal thyroid function was considered as 0.3 mIU/L ≤ TSH ≤ 3.6 mIU/L. The diagnosis of overt and subclinical hypothyroidism respectively was done based on TSH levels higher than 10 and 3.6 mIU/L < TSH ≤ 10 mIU/L [18]. Normal T4 levels were considered between 4.5 and 12.0 μg/dL for normal participants. T4 value lower than 4.5 was one of the additional criteria’ for hypothyroidism patients [18]. The values higher than 40 and 100 IU/mL were considered positive for TPOAb and TGAb, respectively. Diagnosis criteria for Hashimoto thyroiditis included decreased T4 value along with an elevated TSH (Overt and subclinical hypothyroidism patients) and the presence of high serum TPOAb or TGAb concentrations. The patients having overt or subclinical hypothyroidism without positive TPOAb or TGAb were considered as having non-autoimmune hypothyroidism disease. Vitamin D levels lower than 8 ng/mL were considered as severe vitamin D deficiency, 9–15 ng/mL concentrations as mild vitamin D deficiency, higher than 16 to 20 ng/mL concentrations as vitamin D insufficiency and higher than 20 ng/mL concentrations as normal vitamin D level [19].

### Laboratory measurements

Blood samples were taken from all participants after at least 8 h of fasting. T3, Free T4, TSH were measured by Cobas ECLIAs (Roche Diagnostics GmbH, Mannheim, Germany). Thyroid peroxidase antibody (TPOAb) were determined by chemiluminiscenta IMMULITE 2000 XPi (Siemens, Eschborn, Germany). Thyroid globulin antibody (TGAb) levels were analyzed by Enzyme-Linked Immunosorbent Assay (ELISA kit, Diesel). Vitamin D levels were measured by LIAISON vitamin D chemiluminescence immunoassay (DiaSorin, Saluggia, Italy).

### Statistical methods

In order to compare the quantitative continuous variables, ANOVA for parametric data and Man-u withney and Kruskal Wallis for non-parametric data were used. Chi-square test was used to compare discrete data among different groups. A *p*-value of less than 0.05 was considered statistically significant. SPSS v.19 was used for statistical analysis.

## Result

Totally 1138 individuals were studied. Demographic information and biochemical parameters of participants are presented in Table 1. Total vitamin D level of participants was 15.4(8.41–25.87). Male participants had a higher level of vitamin D (*p* = 0.001), based on the Mann–Whitney U test results. While, There wasn’t any significant difference in distribution of male and female participants in study groups (*P* = 0.751). There wasn’t any significant difference in the age of participants of Immune Hypothyroid, Non-Immune Hypothyroid and Control groups (*p* = 0.630).

**Table 1:** Basal characteristic of patients

Characteristic	Immune Hypothyroid	Non-Immune Hypothyroid	control	***P*** Value*
**Number**	633	305	200	–
**Age، years (mean ± s.d.)**	37.48 ± 13.18	36.65 ± 14.56	37.69 ± 15.26	0.630
**Sex (male), n (%)**	146 (23.1)	66 (21.6)	49 (24.5)	0.751
**Vitamin D level, ng/ml (IQR)**	13.22(8.1–24.27)	16(8.43–28.85)	20.4(11.2–29.6)	0.001
**TSH, mIu/l (IQR)**	6.29(3.13–17.75)	5.92(2.54–1.81)	2.2(1.59–2.88)	0.001
**T3, mg/ml (IQR)**	1.67(1.26–2.38)	1.68(1.22–2.15)	1.32(0.74–1.8)	0.001
**T4, mg/ml (IQR)**	9.1(7.06–63.62)	11.1(7.42–80.05)	7.05(0.63–808)	0.001
**TPOAb, IU/mL (IQR)**	14.4(4.69–134.6)	3.59(1.13–16.67)	19.4(13.6–33.55)	0.001
**TGAb, IU/mL (IQR)**	320.7(112.2–733)	10(4.33–15.63)	16.9(9.16–185.95)	0.001

* Kruskal–Wallis one-way analysis of variance. *P* value less than 0.05 is considered significant. Normally distributed variables are shown as mean ± s.d. nonparametric variables are shown as median (IQR). *IQR* Interquartile range. *n* Number, *TSH* Thyroid stimulating hormone. *TPOAb* Thyroid Autoantibodies, *TGAb* thyroglobulin antibodies

**Fig. 2:**
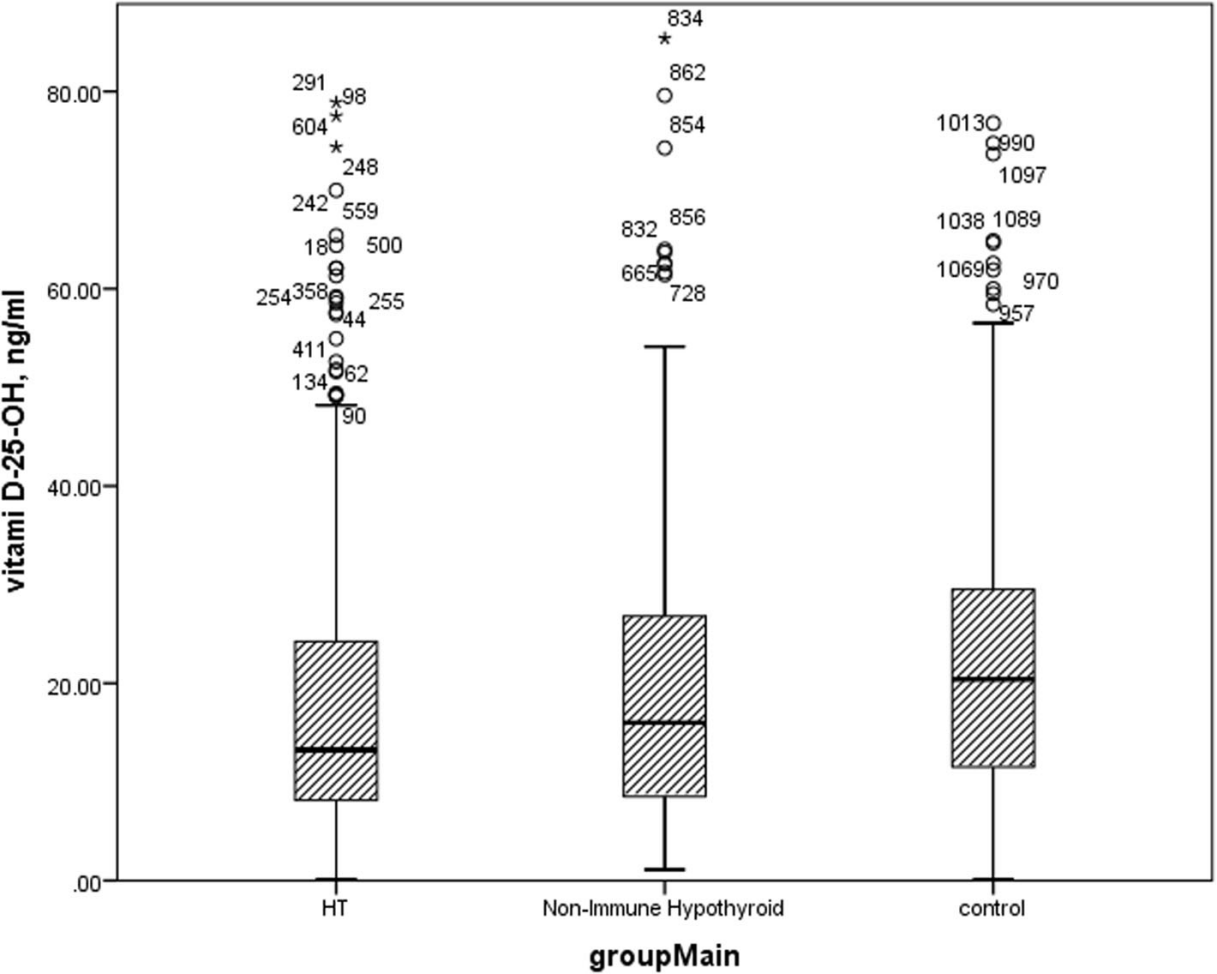
Vitamin D level of study group. Box plot of vitamin D levels in study groups. HT: Hashimoto thyroiditis

Kruskal Wallis test revealed that there was a significant difference in Vitamin D level of study groups. As shown in Fig. 2. Control subjects had significantly higher vitamin D level than both Hashimoto thyroiditis (*p* = 0.001) and Non-autoimmune thyroid disease patients (*p* = 0.001), but there wasn’t any significant difference between vitamin D level of Hashimoto thyroiditis and Non-Immune Hypothyroid patients (*p* = 0.923), as shown in supplementary table 1.

The comparison of study variables between Hashimoto thyroiditis and non-autoimmune hypothyroid groups are provided in Supplementary Table 1. This comparisons didn’t revealed significant difference in vitamin D levels between Hashimoto thyroiditis and non-autoimmune patients (*P* = 0.923). Also the correlations between vitamin D and study variables were evaluated and reported in supplementary file 1.

As shown in Table 2, to investigate the relationship of the thyroid autoimmunity in Hashimoto thyroiditis patients and vitamin D, a chi-square test investigated the distribution of the vitamin D deficiency occurrence in TPOAb+ and TGAb+ patients. The results revealed a significantly higher rate of TPOAb+ in vitamin D deficient patients (*p* = 0.030), however, there weren’t any differences in the occurrence of TGAb+ in vitamin D deficient and sufficient patients (*p* = 0.14).

**Table 2:** Thyroid autoimmunity in Hashimoto thyroiditis patients

	Vitamin D Sufficient (> 20 ng/ml)	Vitamin D Deficient (< 20 ng/ml)	*p value
**TPOAb+, n**	195	422	0.030
**TGAb+, n**	9	34	0.140

* Chi square test. *P* value less than 0.05 is considered significant. *n* number, *TPOAb* Thyroid Autoantibodies, *TGAb* Thyroglobulin antibodies

The spearman test was used to investigate correlation of nonparametric variables including vitamin D and autoantibodies. The results revealed that in Hashimoto thyroiditis patients there was a significant inverse correlation between the vitamin D and TGAb level (*p* = 0.001, r = − 0.261) and a direct correlation of vitamin D with TSH level (*p* = 0.008, r = 0.108). However, there wasn’t any significant correlation between vitamin D and other paraclinical findings). Also, these correlations were not statistically significant in Non-Immune Hypothyroid and Control.

## Discussion

Hypothyroidism is a hypothalamus-pituitary-thyroid imbalance in the central axis and insufficient secretion of the regulatory hormones for thyroid gland [20]. Hypothyroidism may has various reasons that the most common causes are Hashimoto or autoimmune thyroid problems and iodine disorders [21]. Previous studies have been explained the role of vitamin D in preventing autoimmune thyroid disease. Vitamin D prevents aberrant immune responses by modulating the immune cells [22]. However, Vitamin D may affect thyroid function in other ways than modulating the immune system and preventing autoimmune diseases. A limited number of studies were performed in the field of evaluating vitamin D effect in non-immune hypothyroidism disease.

The results of the present research indicated that hypothyroidism patients with or without an immune base, deal with vitamin D deficiency (< 20 ng/mL) more than healthy people. In the study of Evliyaoğlu et al. [23], the patients with < 20 ng/mL vitamin D level was considered as vitamin D deficient and they showed that the prevalence of vitamin D deficiency is more common in people with Hashimoto than normal people. The results of their study were in agreement with those of the present study and other similar researches [16, 17, 24]. In the present study, a remarkable finding was the association between non-autoimmune hypothyroidism and vitamin D deficiency. It means that the vitamin D deficiency level in a patient with non-Hashimoto hypothyroidism was higher than the control group. The relationship between vitamin D deficiency and Hashimoto thyroiditis is well documented. The prevalence of positive TPOAb among people with < 20 ng/mL vitamin D level was more than people with sufficient vitamin D.

Krysiak et al. [25] indicated that daily uptake of 2000 IU and vitamin D can improve the treatment process in women with HT. Of course, the relationship between Hashimoto and vitamin D may also be affected by the patient’s condition. Some scholars believe that there are some differences in the past time since the onset of the disease. Effraimidis et al. [26] stated that there is no relationship between vitamin D level and primary stage of autoimmune thyroid disease. However, our subjects had crossed the initial stage of the disease. Age and sex differences may also be effective. In the present research, the male had a higher level of vitamin D than women.

Colbay et al. showed the negative correlation between TSH and vitamin D level [27]. Zhang et al. [28] indicated that the higher level of vitamin D leads to a reduction of circulated TSH, which are not in agreement with the results of our study. But in non-immune hypothyroidism, vitamin D didn’t correlate with TSH. This shows the diverse probable role of vitamin D deficiency in the pathogenesis of Hashimoto thyroiditis or non-immune hypothyroidism.

The subjects of the present study were under Levothyroxine therapy, as an effective factor on TSH level, interpreting the results based TSH and finding the relationship between vitamin D and thyroid function was not reliable. Barsony et al. [29] demonstrated that the treatment of hypothyroidism with Levothyroxine led to an increase in vitamin D level. While Cayir et al. showed that long-term consumption of levothyroxine may disrupt the concentration of vitamin D [30]. Therefore, there is a complicated relationship between them. Besides, similar clinical and laboratory studies did not address this issue, and they only showed the relationship between Hashimoto thyroiditis and vitamin D. Fournier et al. induced autoimmune thyroid disease on laboratory mice model and showed the positive effect of vitamin D on suppressing of immune system and prevention of thyroid autoimmune damage [31]. However, the present study showed that vitamin D deficiency not only affects the immune system but also have a relationship with the function of the thyroid gland directly.

One of the strong points of the present study is the evaluation of vitamin D level in hypothyroidism patients who their disease wasn’t autoimmune. In the present study, TGAb and TPOAb antibodies were evaluated in thyroid patients, but other factors were not evaluated. The different stage of disease in participants was the limitation of the study. For future researches, we suggest matching factors such as the stage of the disease and the dosage of daily received drugs.

## Conclusion

Finally it can be concluded that vitamin D deficiency may have direct associations with thyroid gland function and indirectly may affect thyroid by modulating immune system. However, further studies are needed to identify the exact molecular mechanism of this hypothesis in non-immune hypothyroidism. Also, screening for vitamin D deficiency may be helpful in all hypothyroid patients.

## Supplementary information

**Additional file 1: Table S1.** Comparison of study variables in Immune vs. Non-Immune Hypothyroid.

## Data Availability

There isn’t any additional data but the data sets are available on the internal medicine department of Jahrom University of medical sciences and would be shared with anyone providing reason for using dataset by contacting the corresponding author.
