# Supplementary material for: Vitamin D deficiency in non-autoimmune hypothyroidism: a case-control study
Source: BMC Endocr Disord. 2020 Mar 20;20:41. doi: 10.1186/s12902-020-0522-9 (PMC7082994; doi:10.1186/s12902-020-0522-9)
Supplement: Supplementary file 1 — Additional file 1: Table S1. Comparison of study variables in Immune vs. Non-Immune Hypothyroid. [file 12902_2020_522_MOESM1_ESM.docx]

**Supplementary file:**

Supplementary table 1. Comparison of study variables in Immune vs. Non-Immune Hypothyroid**.**

| **Characteristic** | **Immune Hypothyroid** | **Non-Immune Hypothyroid** | **p** |
| --- | --- | --- | --- |
| Number | 633 | 305 | - |
| Age، years (mean±s.d.) | 37.48±13.18 | 36.65±14.56 | 0.756 |
| SEX (male), n (%) | 146 (23.1) | 66 (21.6) | 0.625 |
| Vitamin D level, ng/ml (IQR) | 13.22(8.1-24.27) | 16(8.43-28.85) | 0.923 |
| TSH , mIu/l (IQR) | 6.29(3.13-17.75) | 5.92(2.54-1.81) | 0.661 |
| T3,  mg/ml (IQR) | 1.67(1.26-2.38) | 1.68(1.22-2.15) | 0.132 |
| T4,  mg/ml (IQR) | 9.1(7.06-63.62) | 11.1(7.42-80.05) | 0.165 |
| TPOAb, IU/mL (IQR) | 14.4(4.69-134.6) | 3.59(1.13-16.67) | 0.0001 |
| TGAb, IU/mL (IQR) | 320.7(112.2-733) | 10(4.33-15.63) | 0.511 |

* Mann–Whitney U test. P value less than 0.05 is considered significant. Normally distributed variables are shown as mean±s.d. nonparametric variables are shown as median (IQR). IQR: interquartile range. n: number, TSH: thyroid stimulating hormone TPOAb: Thyroid Autoantibodies, TGAb: thyroglobulin antibodies.
